# Preclinical evaluation of noncontact vital signs monitoring using real-time IR-UWB radar and factors affecting its accuracy

**DOI:** 10.1038/s41598-021-03069-2

**Published:** 2021-12-08

**Authors:** Jun-Young Park, Yonggu Lee, Ran Heo, Hyun-Kyung Park, Seok-Hyun Cho, Sung Ho Cho, Young-Hyo Lim

**Affiliations:** 1 Department of Electronics and Computer Engineering, College of Engineering, Hanyang University, 222 Wangsimni-ro, Sungdong-gu, Seoul, 04763 Republic of Korea; 2 Division of Cardiology, Department of Internal Medicine, College of Medicine, Hanyang University, 222 Wangsimni-ro, Sungdong-gu, Seoul, 04763 Republic of Korea; 3 Department of Pediatrics, College of Medicine, Hanyang University, Seoul, 04763 Republic of Korea; 4 Department of Otorhinolaryngology, College of Medicine, Hanyang University, Seoul, 04763 Republic of Korea

**Keywords:** Biomedical engineering, Preclinical research

## Abstract

Recently, noncontact vital sign monitors have attracted attention because of issues related to the transmission of contagious diseases. We developed a real-time vital sign monitor using impulse-radio ultrawideband (IR-UWB) radar with embedded processors and software; we then evaluated its accuracy in measuring heart rate (HR) and respiratory rate (RR) and investigated the factors affecting the accuracy of the radar-based measurements. In 50 patients visiting a cardiology clinic, HR and RR were measured using IR-UWB radar simultaneously with electrocardiography and capnometry. All patients underwent HR and RR measurements in 2 postures—supine and sitting—for 2 min each. There was a high agreement between the RR measured using radar and capnometry (concordance correlation coefficient [CCC] 0.925 [0.919–0.926]; upper and lower limits of agreement [LOA], − 2.21 and 3.90 breaths/min). The HR measured using radar was also in close agreement with the value measured using electrocardiography (CCC 0.749 [0.738–0.760]; upper and lower LOA, − 12.78 and 15.04 beats/min). Linear mixed effect models showed that the sitting position and an HR < 70 bpm were associated with an increase in the absolute biases of the HR, whereas the sitting position and an RR < 18 breaths/min were associated with an increase in the absolute biases of the RR. The IR-UWB radar sensor with embedded processors and software can measure the RR and HR in real time with high precision. The sitting position and a low RR or HR were associated with the accuracy of RR and HR measurement, respectively, using IR-UWB radar.

## Introduction

Real-time monitoring for vital signs such as respiratory rate (RR), oxygen saturation, heart rate (HR), blood pressure (BP) and body temperature is fundamental in many clinical contexts. Numerous studies have reported that changes in vital signs can predict health deterioration events in advance^1–3^. Existing vital sign monitors require contact sensors, which may cause not only discomfort and irritation on the skin^4^ but also transmission of contagious diseases^5^. The recent global COVID-19 pandemic has raised issues related to the in-hospital transmission of infectious diseases between medical staff and patients^6^, and an urgent need for the development of noncontact vital sign monitoring technologies has emerged.

Radar systems exploit electromagnetic waves to detect and range targets. Impulse-radio ultrawideband (IR-UWB) radar occupies a wide range of frequencies > 500 MHz and uses pulse wave signals in the time domain. IR-UWB radar penetrates materials effectively and can be implemented with simple hardware; it is also highly resistant to multipath signal propagation^7^. In contrast to camera-based photoplethysmography, radar can recognize targets through various obstacles, such as fog, dust, clothes and curtains, and is highly resistant to noise from ambient lights. Therefore, in previous studies, we achieved successful monitoring of HR and RR using IR-UWB radar in patients with cardiovascular diseases in a preclinical setting^8^.

In previous studies, we measured HR and RR simultaneously by targeting the neck area^8^. The signals from carotid pulsation on the surface of the neck were more prominent than respiration signals by a sufficiently wide margin to distinguish the two, in contrast to heartbeat signals on the anterior chest, which were difficult to distinguish from respiration signals. However, this method requires the radar sensor to be placed a short distance (30 cm) from the neck and aimed with pinpoint accuracy at the carotid pulsation, which may be a major limitation for a noncontact vital sign monitor given that careful positioning of the device at close range may pose just as much risk as direct contact from the perspective of contagious disease prevention. Moreover, in previous studies, we measured HR and RR only in a supine position, which may be impractical in outpatient clinic settings and ignored the effect of posture on the accuracy of radar measurements. Additionally, the demographic and anthropometric features that might influence the radar measurement results have never been studied. Finally, a radar senor meant for widespread clinical use should be a standalone device that monitors RR and HR in real time and has a user-friendly interface.

Here, we developed an IR-UWB radar sensor that features embedded processors and software and can perform simultaneous real-time HR and RR monitoring from the chest at a comfortable distance. We evaluated the accuracy and reliability of HR and RR measurements obtained using the new IR-UWB radar sensor and investigated the factors affecting the accuracy of the radar measurements, including patient posture.

## Subjects and methods

### Participants

Volunteer participants were recruited among patients who visited the outpatient clinic of the Department of Cardiology at Hanyang University Medical Center. Patients older than 18 years with normal sinus rhythm after initial clinical assessments were included. Patients with severe or uncontrolled symptoms of cardiovascular diseases were excluded. Demographic information, anthropometric measurements, medical history and standard 12-lead electrocardiography (ECG) were obtained before the radar measurements. All procedures in this study protocol adhered to the ethical principles of the Declaration of Helsinki. Written informed consent was provided by all patients before they were enrolled in the study. The Institutional Review Board of Hanyang University Hospital reviewed and approved the study protocols and monitored the study processes (IRB No. 2017-05-004-017).

### Experimental conditions

**Figure 1:**
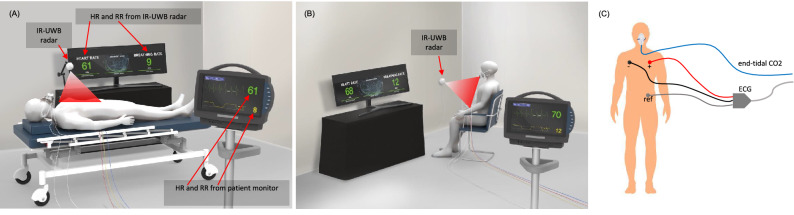
The graphical description of the experiment environment. HR and RR were measured simultaneously using an IR-UWB radar sensor and a patient monitor in both supine (**A**) and sitting (**B**) positions. ECG and capnometry was obtained using the patient monitor to determine the reference values of HR and RR respectively. ECG and the capnometry setup is described in (**C**). The measurements of HR and RR were conducted for 2 min in each posture. The radar sensor was placed on a tripod at a 1.5 m distance, in the sitting position, and suspended at 1.5 m above in the supine position, directing to the anterior chest wall.

Participants rested for 5 min while sitting in a chair in an examination room before the HR and RR were measured. First, the participants changed to a supine position and rested for 1 min while minimizing their movements; measurements were then obtained in that position for 2 min. Next, participants rested for 1 min in a sitting position and were then recorded for 2 min in that position. Participants were asked to minimize their body movements during the measurements (Fig. 1).

In the supine position, the radar sensor was perpendicularly suspended at a diagonal distance of 1.5 m above the chest wall and directed toward the chest during the measurements. In the sitting position, the radar sensor was placed in front of the participants at a distance of 1.5 m from the chest wall and was once again directed toward the chest during the measurements. Signals were processed by a chip embedded in the radar sensor, and the estimates of RR and HR were sent to a Raspberry Pi for transmission to a server through the internet. Simultaneously the radar measurements, a patient monitor (BSM-6701 K, Nihon Kohden, Shinjuku City, Tokyo, Japan) was used to record the HR by ECG and the RR by capnometry measuring exhaled CO_2_ levels to determine reference values for the corresponding vital signs. The RR and HR were calculated onboard by the patient monitor, recorded in a memory card with a timestamp and then synchronized with the data from the radar sensor before analysis.

### Radar setup and signal process

A commercially available radar sensor (XK300-VSA, Xandar Kardian, Delaware, USA) was used for the IR-UWB radar measurements. The frequency range of the radar sensor was 6.5–8.0 GHz, and the transmission power was < 41.3 dBm/MHz. The antenna pair included 1 transmitter and 1 receiver.

**Figure 2:**
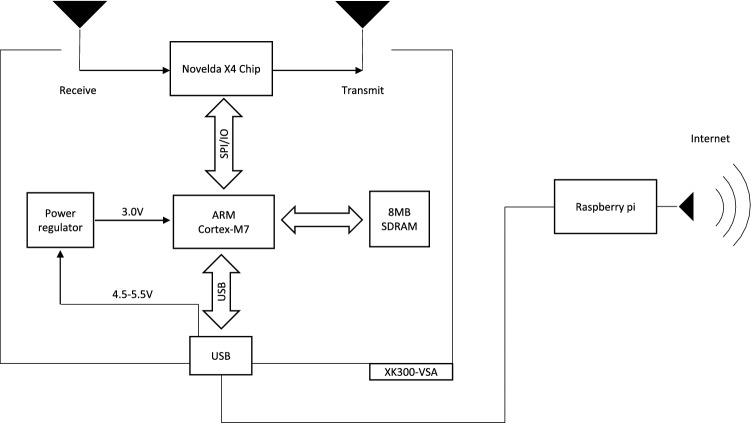
Hardware diagram of XK300-VSA. The ARM Cortex-M7 controls the radar chip to transmit and receive the radar signal and then computes the RR, HR and relative movement index. After the whole procedure, RR, HR and relative movement index are transmitted to the Raspberry pi through USB to be sent to the server for archive.

The hardware structure of the XK300-VSA is described in Fig. 2. The microcontroller unit (MCU), an ARM Cortex-M7, operated a radar chip that transmitted a pulse of a 7.25 GHz sine-wave carrier, which was modulated with a Gaussian amplitude, received the reflected radar signals and stored them in synchronous DRAM (SDRAM). Then, the MCU obtained the radar matrix from the SDRAM and processed it. The results were sent to the Raspberry Pi (Raspberry Pi 3 Model B, Raspberry Pi Foundation, Cambridge, England, UK) through a USB cable and then sent to the server through the internet.

The software structure of the embedded processor of the radar device had 3 signal processing modules for the estimation of RR, HR and the relative movement index. The algorithms for signal processing and communication were encoded using C# language. The MCU in the radar device assessed occupancy using the relative movement index. The relative movement index, *m(t),* was used as a parameter to decide whether to process the data and to estimate the RR and HR; this index was defined as the sum of all differences between the previous and current raw radar signals as follows:

$$m\left( t \right) = \mathop \sum \limits_{\;\;} \left| {r\left( {t,\tau } \right) - r\left( {t - 1,\tau } \right)} \right|$$

where $$\;r\left( {t,\tau } \right)\;$$ is the received signal at slow time $$ t$$ and fast time $$\tau $$. The fast time was determined by distance, and the slow time was determined by time. When a patient was within the measurement range of the radar sensor, the MCU measured the participant’s movements and quantified them to calculate the relative movement index. Then, when the amount of movements was sufficiently small according to the relative movement index criteria, the radar sensor produced the estimates of RR and HR and transmitted the data to the server. The radar sensor was activated at a rate of 10 frames per second (FPS), and RR and HR estimates were produced every second. Because the data were transferred to the server through the internet using a public wireless network provided in the examination room, the data flow rate was set to 0.36/s.

If the participant was resting without any motion, it was possible to hypothesize that the only movements were caused by respiration and heartbeat. The movement can be modeled as follows:

$${\text{p}}\left( t \right) = {p_0} + {m_r}sin\left( {2\pi {f_r}t} \right) + {m_h}sin\left( {2\pi {f_h}t} \right) $$

where p(t) indicates movement, p_0_ is the initial position of the participant, f_r_ and f_h_ indicate the frequency of respiration and heartbeat, respectively, and m_r_ and m_h_ indicate the magnitude of respiration and heartbeat, respectively. The radar signal reflected and received by the target also vibrated according to the movement, and the movement could be observed by tracking the peak of the received signal. However, tracking the peak of the signal did not provide sufficient resolution to observe respiration and heartbeat. This required tracking of the phase differences of the received signal because the phase reacts sensitively to differences in the signal. The basic mechanism for obtaining respiration and heartbeat using IR-UWB radar has been thoroughly described previously^9,10^.

**Figure 3:**
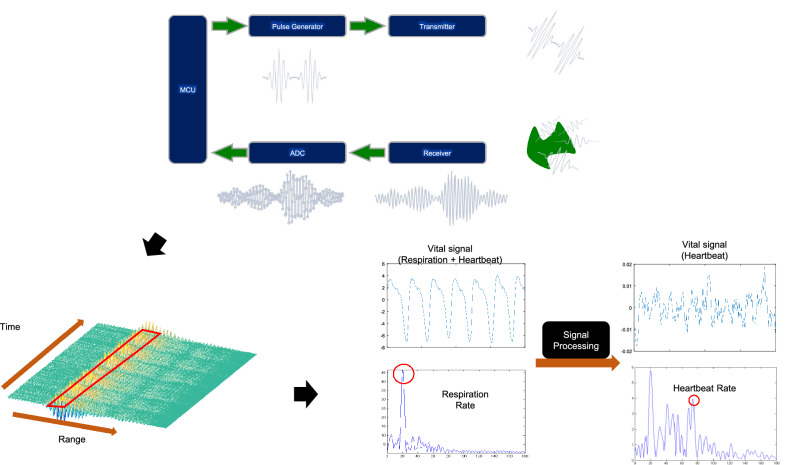
Data flow from the IR-UWB radar sensor. Radar transmits electromagnetic waves to target and receives the reflected signals. The ADC inside the signal processing unit samples and quantizes received signals to convert the analog signals to the digital signals and save it in the buffer. The recorded radar signals form radar cube, from which the target location can be estimated to extract the vital signals. The extracted vital signals are sent to the filters and processed using the fast Fourier transformation to produce RR and HR.

The RR and HR measurement process is described in Fig. 3. The received radar signals were digitized by the chip of the radar device and stored in SDRAM. Because of the limitation in memory capacity, the radar signals were recorded in 20-s increments. The raw signals were sent to a recursive moving target indication (MTI) filter to remove the clutters that consisted of unwanted and usually constant signals to locate the target and identify the vital signs. After the MTI filter was applied, the relative movement index was calculated. Once the movements were reasonably minimized, the target location was identified. The target location was determined in the range with the largest signal variation in the 20 s of recorded signals. The vital signs were obtained from the signals in the target range by scraping the data along the slow time axis. Because HR occupies higher frequency ranges and has lower power levels than RR, RR and HR could be separated using a bandpass filter. The filter range for RR was 0.08–1.00 Hz, and that for HR was 0.83–2.17 Hz. The signals for RR and HR were passed through their respective filters, and fast Fourier transform (FFT) was simultaneously applied to the signals, providing estimates of RR and HR.

### Statistical analysis

Baseline data, including age, sex, height, weight, body mass index (BMI), RR and HR, are presented as the mean ± SD or numbers (%). The agreement of RR and HR between IR-UWB radar and the conventional patient monitors was evaluated using concordance correlation coefficients (CCCs), intraclass correlation coefficient (ICC), and the levels of bias and the limits of agreement (LOAs) between the radar and the conventional patient monitors were evaluated using Bland–Altman (BA) plots. The presence of systematic biases in the measurements of the RR and HR was evaluated using a linear regression analysis between their average values of and the biases between them.

Bias, mean bias, absolute bias and absolute mean bias were defined as follows:

$$ {\text{Bias }}\left( {\text{for individual measurement}} \right) = {V_k}{\text{RD\;}}-{\text{\;}}{V_k}{\text{CM}}$$

$$ {\text{Absolute bias}} = \left| {{V_k}{\text{RD\;}}-{\text{\;}}{V_k}{\text{CM}}} \right| $$

$${\text{\;Mean bias}} = \frac{1}{{\text{N}}}*\mathop \sum \limits_{k = N}^{k = 1} \left( {{V_k}{\text{RD\;}}-{\text{\;}}{V_k}{\text{CM}}} \right)$$

$$ {\text{Absolute mean bias}} = \left| {\frac{1}{{\text{N}}}*\mathop \sum \limits_{k = N}^{k = 1} \left( {{V_k}{\text{RD\;}}-{\text{\;}}{V_k}{\text{CM}}} \right)} \right|$$

where ${V_k}{\text{RD}}$ and ${V_k}{\text{CM}}$ indicate the *k*th measurement value obtained from the radar and the conventional monitors, respectively, and N indicates the number of measurements from one participant.

The absolute biases in the measurements of RR and HR between the radar and conventional patient monitors were compared in terms of sex, age (< 50 vs. ≥ 50 years), BMI (< 20 vs. 20–24.9 vs. ≥ 25 kg/m^2^) and posture (sitting vs. standing) using the Mann–Whitney U test. The relationships of the absolute biases in the RR or HR measurements with the reference RR or HR were identified using linear regression analysis with a restrictive cubic spline fit. Using these restrictive cubic spline models, cutoff points for the RR and HR in mixed linear effect models were decided.

The influence of demographic information, anthropometric factors and posture on the absolute bias in RR and HR measurements was evaluated using linear mixed effect models. In the models, the variances resulting from individual participant identity were considered a random effect, and the variances resulting from differences in sex, age, BMI, posture and RR or HR were considered fixed effects.

All statistical analyses were performed using the statistical software R 4.2 (R Core Team, R Foundation for Statistical Computing, Vienna, Austria) and RStudio 1.3.969 (RStudio Team, Rstudio, BPC, Boston, MA, US) and R packages including lmerTest, car, descr and rms. A *p* value < 0.05 was considered significant.

## Results

A total of 50 participants were included in this study. The baseline characteristics of the participants are summarized in Table 1. The mean age was 44.3 ± 14.6 years, and 52% of the participants were male. The mean RR was 15.7 ± 4.4 breaths/min, and the mean HR was 72.2 ± 11.2 bpm.

**Table 1:** Baseline characteristics of the participants.

	N = 50
Age (year)	44.30 ± 14.61
Sex (male)	26 (52.0%)
Height (m)	1.68 ± 0.09
Weight (kg)	66.14 ± 11.91
Body mass index (kg/m^2^)	23.57 ± 4.05
**Underlying disease**	
Hypertension	
Diabetes	
Dyslipidemia	
CAD	
HF	
PAD	
**Respiratory rate (breaths/min)**	15.69 ± 4.38
Supine	15.60 ± 4.39
Sitting	15.76 ± 4.67
**Heart rate (bpm)**	72.23 ± 11.23
Supine	70.82 ± 11.26
Sitting	73.72 ± 11.86

Data are described as the mean ± SD or N (%).

**Figure 4:**
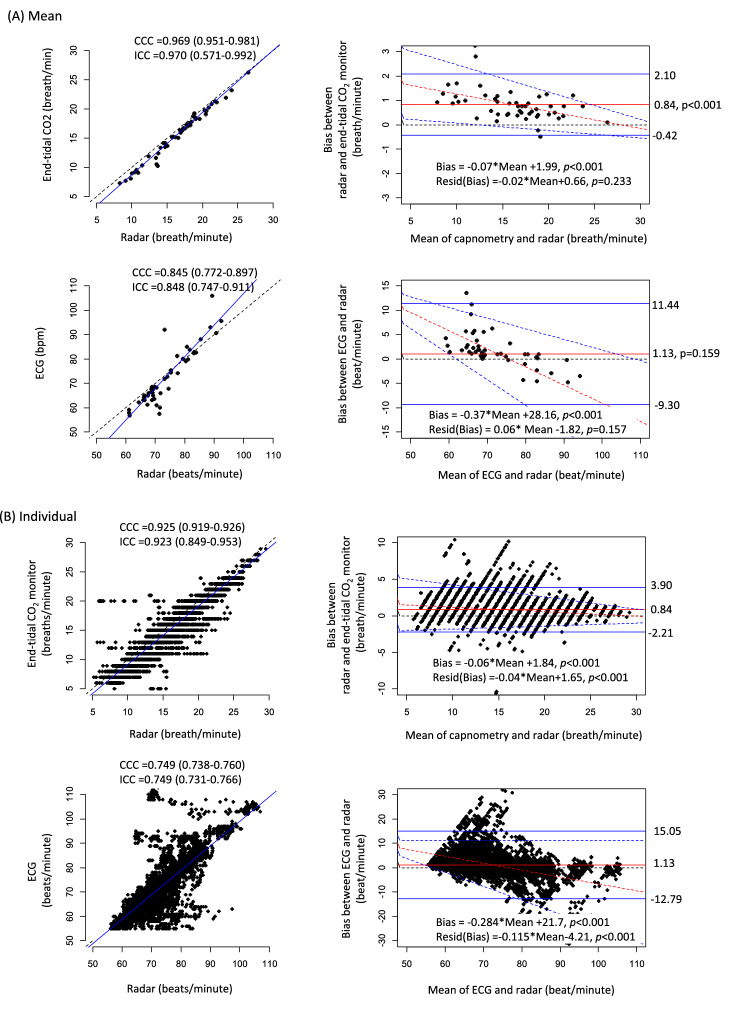
Agreements between RRs and HRs measured using IR-UWB radar and those measured using the conventional patient monitor. (**A**) Agreements of mean RR and HR. (**B**) Agreements of individual RR and HR. The blue solid lines in scatter plots indicates estimates of linear regression models. The blue solid lines in Bland–Altman plots indicate 95% upper and lower LOAs and the red solid lines indicate the mean biases. The red broken lines represent the estimates of linear regression models between the bias and the mean values and the blue broken lines represent the 95% prediction intervals of the biases corresponding to the mean values. *Resid(Bias)* residuals of biases between radar and the conventional monitors.

The levels of agreement between measurements from the radar sensor and those from the conventional monitor were analyzed using CCC, ICC and BA plots. The mean RRs measured for 2 min using radar highly agreed with those measured simultaneously using capnometry (CCC 0.969; 95% CI 0.951–0.981). The BA plot showed that the mean bias of the mean RRs between the 2 methods was 0.84 breaths/min and that the width of the LOAs was 2.52 breaths/min. The bias of the RR gradually decreased as the mean RR increased. The concordance level of the mean HRs between radar and ECG was lower than that of the mean RRs between radar and capnometry (CCC 0.845; 95% CI 0.772–0.897), and the systematic bias of the mean HR was greater than that of the mean RR (Fig. 4A and Table 2). The concordance levels of the mean RR and the mean HR were lower, and the bias of the mean RR and mean HR between radar and the conventional monitors was greater in the sitting position than in the supine position (Table 2).

**Table 2:** Agreements of RR and HR between radar and the conventional monitor according to the postures.

	CCC (95% CI)	ICC (95% CI)	Mean bias (95% CI)	Lower LOA	Upper LOA	Beta, *p*-values*
**Means**						
RR						
all	0.969 (0.951–0.981)	0.970 (0.571–0.992)	0.837 (0.659–1.015)	− 0.42	0.21	− 0.071, < 0.001
Supine	0.970 (0.952–0.981)	0.971 (0.488–0.992)	0.862 (0.694–1.029)	− 0.32	2.04	− 0.045, < 0.001
Sitting	0.958 (0.932–0.975)	0.959 (0.825–0.984)	0.802 (0.524–1.082)	− 1.17	2.77	− 0.105, < 0.001
HR						
all	0.845 (0.772–0.897)	0.848 (0.747–0.911)	1.070 (− 0.397–2.537)	-9.30	11.44	− 0.372, < 0.001
Supine	0.819 (0.724–0.883)	0.822 (0.703–0.896)	1.613 (0.022–3.204)	− 9.64	12.86	− 0.338, < 0.001
Sitting	0.751 (0.622–0.841)	0.755 (0.605–0.853)	0.322 (− 1.697–2.341)	− 13.95	12.58	− 0.361, < 0.001
**Individual**						
RR						
all	0.923 (0.919–0.926)	0.923 (0.849–0.953)	0.843 (0.801–0.883)	− 2.21	3.90	− 0.067, < 0.001
Supine	0.941 (0.937–0.945)	0.941 (0.826–0.971)	0.882 (0.849–0.914)	− 1.56	3.33	− 0.039, < 0.001
Sitting	0.903 (0.896–0.910)	0.903 (0.848–0.933)	0.800 (0.752–0.849)	− 2.80	4.40	− 0.086, < 0.001
HR						
all	0.749 (0.738–0.760)	0.749 (0.731–0.766)	1.129 (0.941–1.316)	− 12.80	15.05	− 0.284, < 0.001
Supine	0.787 (0.774–0.799)	0.787 (0.755–0.814)	1.549 (1.381–1.717)	− 10.88	13.98	− 0.278, < 0.001
Sitting	0.706 (0.688–0.724)	0.707 (0.686–0.726)	0.676 (0.469–0.883)	− 14.64	15.99	− 0.288, < 0.001

*RR* respiratory rate, *HR* heart rate, *CCC* concordance correlation coefficient, *LOA* limit of agreement.

*Beta coefficients and *p*-values were derived from a linear regression analysis between the biases and the average between radar and the conventional monitor.

The individual RRs measured using radar highly agreed with those measured using capnometry (Fig. 4B). The BA plot showed that the mean bias of individual RRs between radar and capnometry was as small as 0.84 breaths/min. However, the width of the LOAs was 6.11 breaths/min, approximately 38% of that of the mean RRs. The bias of individual RRs gradually decreased as the individual RRs increased, but the changes were only minimal (1.48 [1.29–1.73] breaths/min for the entire RR range). The concordance levels of individual HRs between radar and ECG were lower than those of individual RRs between radar and capnometry (Table 2). The mean bias of the individual HRs was small but significant, and the width of the LOAs was 27.8 bpm. The bias of the individual HRs decreased as the HRs increased, more rapidly than the biases of the individual RRs did (Fig. 4B; Table 2). In general, the concordance levels between the 2 methods were lower in the sitting position than in the supine position for both individual RRs and individual HRs (Table 2).

**Figure 5:**
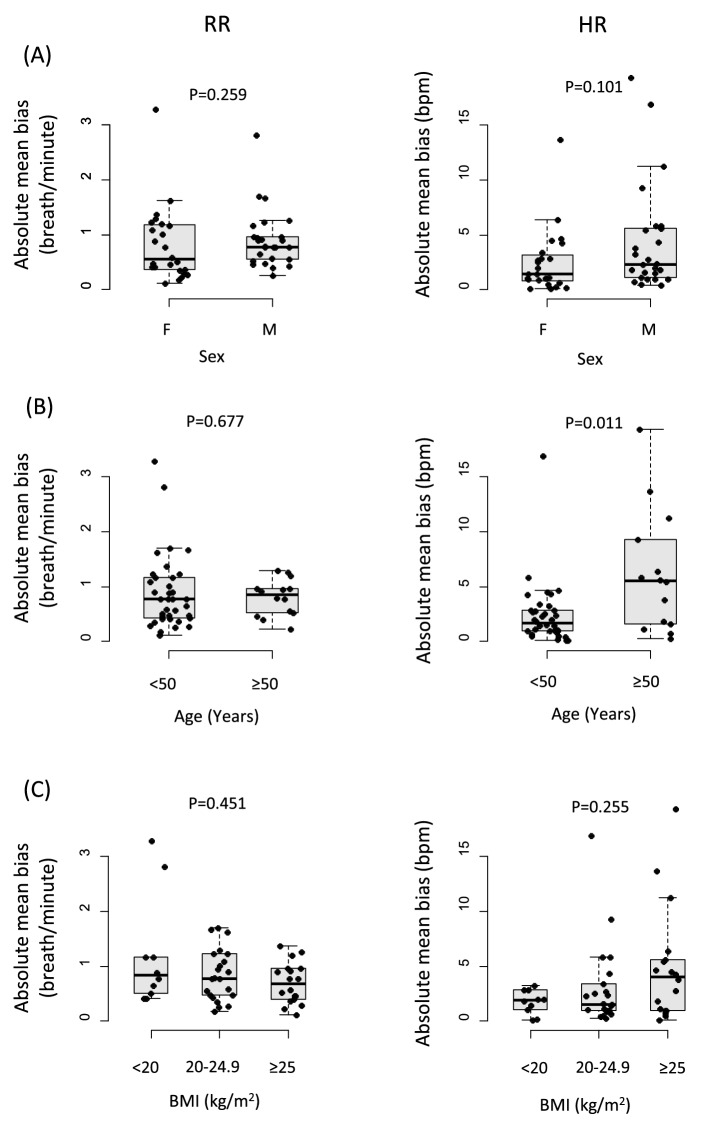
Comparisons of mean absolute biases between the radar and the conventional monitor according to sex, age and BMI. (**A**–**C**) represent the absolute bias levels in RRs and HRs according to sex, age and BMI, respectively. Absolute biases of RRs were lower in the BMI ≥ 25 kg/m^2^, whereas absolute biases of HRs are greater in male, in patients ≥ 50 years and in those with BMI ≥ 25 kg/m^y^ in both the supine and the sitting positions.

The absolute biases of the individual RRs gradually decreased until the individual RRs reached 18 breaths/min and then plateaued afterward, whereas the absolute biases of the individual HRs gradually decreased, reached troughs between 70 and 80 bpm, and then increased afterward (Supplementary Fig. 1). The absolute biases of the individual RRs were lower in participants with BMI ≥ 25 kg/m^2^ but did not significantly differ with sex and age. The absolute biases of the individual HRs were higher in males, participants ≥ 50 years old, and those with a BMI ≥ 25 kg/m^2^ (Supplementary Fig. 2). In contrast, the absolute mean biases of the RRs did not differ with sex, age or BMI. The absolute mean biases of the HRs also did not differ with sex and BMI, although they were higher in participants  ≥ 50 years old (Fig. 5).

To determine how significantly posture, age, sex, body size and RR/HR influenced the accuracies of the radar measurements, linear mixed effect models were used (Table 3). Both univariate models and the multivariate model showed that the sitting position and RR ≥ 18 breaths/min were associated with absolute biases in the RR measurements. The sitting position increased the absolute bias in RR by 0.19 breaths/min, and a mean RR < 18 breaths/min increased it by 0.86 breaths/min. However, unlike the simple comparisons between groups, age ≥ 50 years, male sex and a high BMI were not associated with absolute biases in the RR measurements. In the multivariate model without RR (Model 2), only the sitting position was associated with increased absolute biases in the RR. Univariate models showed that age ≥ 50 years and HR < 70 bpm were associated with increased absolute biases in the HR measurements. However, the multivariate model showed that sitting position and a mean HR < 70 bpm increased the absolute bias of the HRs by 0.31 bpm and 1.07 bpm, respectively, and age, sex, BMI and HR ≥ 80 bpm were not associated with the absolute biases in HR measurements (Table 3). In the multivariate model without HR (Model 2), none of the covariates was significantly associated with increased absolute biases in the HR.

**Table 3:** Determinants of the absolute bias levels.

Models	Variables	Univariate		Multivariate model 1		Multivariate model 2^a^	
		Estimate (95% CI)	*p-*value	Estimate (95% CI)	*p-*value	Estimate (95% CI)	*p-*value
Absolute bias of RR	Posture supine vs. sitting	0.104 (0.039 to 0.168)	0.002	0.186 (0.122 to 0.251)	< 0.001	0.103 (0.039 to 0.168)	0.002
	age < 50 vs. ≥ 50 years	0.115 (− 0.241 to 0.472)	0.529	0.130 (− 0.261 to 0.521)	0.515	0.189 (− 0.211 to 0.589)	0.371
	Sex M vs. F	0.056 (− 0.266 to 0.377)	0.736	− 0.071 (− 0.399 to 0.257)	0.671	0.036 (− 0.300 to 0.371)	0.839
	BMI (kg/m^2^)	− 0.015 (− 0.055 to 0.025)	0.468	− 0.004 (− 0.047 to 0.038)	0.837	− 0.024 (− 0.067 to 0.019)	0.290
	RR < 18 breath/min	0.809 (0.700 to 0.918)	< 0.001	0.864 (0.753 to 0.974)	< 0.001	–	–
Absolute bias of HR	Posture supine vs. sitting	0.158 (− 0.073 to 0.389)	0.181	0.308 (0.063 to 0.553)	0.014	0.157 (− 0.073 to 0.389)	0.181
	age < 50 vs. ≥ 50 years	3.201 (0.983 to 5.419)	0.007	0.051 (− 0.031 to 0.132)	0.229	0.056 (− 0.027 to 0.134)	0.180
	Sex M vs. F	1.800 (− 0.291 to 3.891)	0.098	1.429 (− 0.702 to 3.559)	0.195	1.588 (− 0.459 to 3.635)	0.144
	BMI (kg/m^2^)	0.225 (− 0.036 to 0.485)	0.098	0.102 (− 0.196 to 0.400)	0.506	0.095 (− 0.192 to 0.382)	0.528
	HR < 70 bpm	0.932 (0.493 to 1.371)	< 0.001	1.074 (0.616 to 1.532)	< 0.001	–	–
	≥ 80 bpm	0.316 (− 0.282 to 0.914)	0.301	0.223 (− 0.385 to 0.831)	0.472	–	–

Linear mixed effect models were performed with individuals as the random term were used to estimate the effects of the factors.

*BMI* body mass index, *M* male, *F* female, *HR* heart rate, *RR* respiratory rate.

^a^Multivariate model 2 = multivariate model 1 minus RR (or HR).

## Discussion

In this study, we successfully monitored the RR and HR in 50 patients visiting the cardiology department using an IR-UWB radar sensor with embedded processors and software; we then analyzed the accuracy of the measurements and identified the determinants of the absolute biases between the measurements from the radar and those from the conventional vital sign monitors. The agreement of the mean RRs and individual RRs between radar and the conventional monitors was substantial, whereas that of the mean HRs and individual HRs between the two methods was modest. The agreement levels in both the RR and HR measurements were lower in a sitting position than in a supine position, but the influences of posture on the bias and agreement were greater for the HR measurements than for the RR measurements. A sitting position and an RR < 18 breath/min and a sitting position and an HR < 70 bpm were associated with a lower accuracy of radar measurements for the RR and HR, respectively.

### Meaning of this study: what is new, and what is the value of the content?

As interest in noncontact vital sign monitoring technologies has increased with the recent global pandemic, many noncontact vital sign monitoring sensors have been developed. IR-UWB radar is a promising technology, given its safety, good penetration power, high resolution and the independence of its physical properties from luminance, opacity, air conditions and clothing^1^. Through our previous studies, we have made progress in developing radar technology for measuring vital signs. We developed a method to measure heartbeat-to-heartbeat (R-R) intervals and to detect arrhythmia during a short period of breath holding^11^, followed by a method to measure the R-R intervals simultaneously with RR from the carotid artery area^10^. We also reported a method to monitor HR and RR simultaneously in infants^12^ and in patients under nocturnal polysomnography^13^. However, for a radar sensor to be applicable in present-day clinical practice, real-time monitoring with a ready-to-use single-unit device and a user-friendly interface and sufficient testing in multiple clinical scenarios are as necessary as high accuracy and reliability. In this study, we designed an internet-connected vital sign-monitoring radar sensor with embedded data processing units and software that could measure RR and HR without external equipment or software. Using this radar sensor, we monitored the RR and HR in real time from a comfortable distance from patients visiting a cardiology clinic. This could be an important step toward a commercially available radar sensor for vital sign monitoring.

### Selling point of this study

In this study, to explore factors influencing the accuracy of the radar measurements, we included 50 patients aged between 20 and 80 years who visited a cardiology clinic. This is the largest sample size among the articles that we have published regarding radar vital sign measurement technologies. We also conducted the measurements in the 2 postures most frequently applied to actual patients, namely, supine and sitting; we found that posture had a strong influence on the accuracies of the radar measurements.

In previous studies, we used manual counting^10^, impedance pneumography^12^ and a chest belt^13^ to measure RR, which may result in errors related to human mistakes or minor body movements. In this study, we used a capnometry device as the conventional monitor for RR measurements, which is known to be the most accurate and well standardized method for measuring the RR^14^.

### Accuracy of HR measurements

In the results, the accuracy of the radar sensor in measuring the RR was sufficiently high to allow its use in clinical practice. The mean bias in the average and individual RRs was < 1 breath/min, and the distances between the LOAs for the mean and individual RRs were 2.5 and 6.1 breaths/min, respectively, which are comparable to or even less than the mean biases and the distances between LOAs reported in studies comparing impedance pneumography and capnometry^15,16^. The accuracy of the HR measurements was lower than that of the RR measurements. The CCCs and ICCs were approximately 0.7–0.85, and the distances between the LOAs were 20.7 bpm (28.7% of the mean HRs). A pulse oximeter, the device most frequently used for HR measurements other than ECG in clinical settings, has been reported to have distances of 10.4 bpm between the LOAs (half of our reported range) for HR measurements^17^, although its accuracy is known to decrease under some conditions, including for patients with dark skin, hypoxia, low blood pressure and rapid HR^18^, which may not influence the radar accuracy. The accuracy of radar in measuring the HR should be compared with that of other noncontact devices. To date, several noncontact vital sign monitors have been introduced, including devices that employ photoplethysmography with a camera and ambient light, thermography, laser Doppler vibrometers and continuous-wave Doppler radar, but few of them have been validated as independent devices capable of real-time monitoring in a clinical setting^19–23^. Moreover, the accuracy of HR measurements using radar is still comparable to that of other noncontact HR monitoring methods, including camera-based photoplethysmography^19,24^ and continuous wave Doppler radar^25^.

### Factors influencing the measurement biases

The posture consistently impacted the accuracy of both RR and HR measurements with the radar sensor. The CCCs and ICCs were consistently higher by 0.012–0.068 (1.2–8.3%), and the distances between the LOAs were also smaller in the supine position than in the sitting position. The mixed linear effect models showed increased absolute biases by approximately 0.19 breaths/min and 0.31 bpm in the sitting position. Studies have shown that forward movements of the chest wall are actually greater in a sitting position than in a supine position^26^; thus, the radar signals would have been stronger and more recognizable in a sitting position. However, a sitting position may be more susceptible to small, unnoticeable body movements because some tension must be maintained between the extensor and flexor muscles in that position^25^. In addition, the larger anterior–posterior movements of the chest wall in the sitting posture would definitely complicate the accurate measurement of heartbeats. The fact that the positional differences in the accuracy indices and the coefficient of the sitting position in the linear mixed effect models were greater in the HR measurements than in the RR measurements may also support this idea. The association between high RRs and low absolute biases in individual RRs may reflect remaining body movements during radar measurement, which could be more easily detected by radar at a low RR regardless of posture. Similarly, the absolute biases in individual HRs increased at low HR levels, which may also reflect remaining noises measured at frequencies in the HR range.

Although the absolute biases in the individual RRs or HRs appeared to be different according to BMI, age or sex, the absolute mean bias in the RR or HR was much less influenced by these clinical factors. The apparent differences in the absolute biases theoretically depend on the number of samples from each participant, which is also linked to the mean RR/HR of the participant, not to the clinical features. Consequently, age, sex, and BMI did not have significant associations with the absolute biases in the individual RRs/HRs in the mixed linear models, in which the variance within each participant was set to be the random effect. Although 50 participants may be sufficient to test the variances of 5 variables, larger numbers of participants would be desirable to increase the power of the analyses.

The RR and HR in the linear mixed effect models may not provide any prediction for the absolute biases in the radar RR/HR measurements because the RR/HR cannot be known before the radar measurements. However, the purpose of these models was not to predict the level of bias in radar measurements but to identify factors determining the amount of error in radar measurements. Moreover, few studies have reported on the patient factors affecting the accuracy of radar vital sign measurements, including postures. Therefore, the results from the models could still provide useful information for physicians to interpret the radar measurements and improve the performances of their biomedical radar sensors.

### Direction of future development

The accuracy of radar-based HR measurements should be improved. Because the influence of posture on accuracy is substantial, it may be useful to consider posture as a variable during signal processing to develop optimized HR estimating algorithms that can be applied to each body position. Additionally, applying an adaptive bandpass filter is considered to improve the accuracy because there could be distortions on the margin of the bandpass filter, causing errors. Moreover, to enable more accurate monitoring and measurement of the HR, further research should be undertaken to enhance the signal-to-noise ratio of the radar signal by utilizing multi-input/multioutput radar. By applying beamformers, it would be possible to narrow the beamwidth and eliminate unwanted signals from locations other than the chest wall.

## Limitations

First, although we tested the accuracy of real-time RR and HR measurements using radar in patients with cardiovascular diseases, the patients were still asked to minimize their movements. To be competitive in the market as a vital sign monitor, a device should guarantee sufficient measurement quality even if there are some body movements. Therefore, data processing algorithms less susceptible to random body movements are still desired. Second, we measured radar signals in 50 patients with a recording duration of 2 min per body posture. The accuracy levels in the results represent the performance of the devices in similar circumstances only. Third, although we measured vital signs using the radar sensor in supine and sitting positions, in most clinical situations, patients require continuous vital sign monitoring on a bed. Therefore, the accuracy of the radar measurements for the RR/HR should be evaluated in the various body postures possible on the bed, including recumbent, left and right decubitus and prone. For radar sensors to gain acceptance in clinical practice, clinical studies involving a larger number of patients tested for longer measurement durations in more varied body postures may be desired.

## Conclusion

The real-time measurement of the RR and HR using our radar sensor with embedded processors and software could be accurate in a clinical setting. The accuracy of the RR and HR measurements may be reduced with a sitting position, a low RR of < 18 breaths/min and a high HR of < 70 bpm. Additional improvements in HR measurement accuracy are desired to promote the clinical use of the radar sensor as a real-time vital sign monitor.

## Supplementary Information

Supplementary Information.
