# Supplementary material for: Preclinical evaluation of noncontact vital signs monitoring using real-time IR-UWB radar and factors affecting its accuracy
Source: Sci Rep. 2021 Dec 8;11:23602. doi: 10.1038/s41598-021-03069-2 (PMC8655004; doi:10.1038/s41598-021-03069-2)
Supplement: Supplementary file 1 — Supplementary Information. [file 41598_2021_3069_MOESM1_ESM.pptx]

## Slide 1
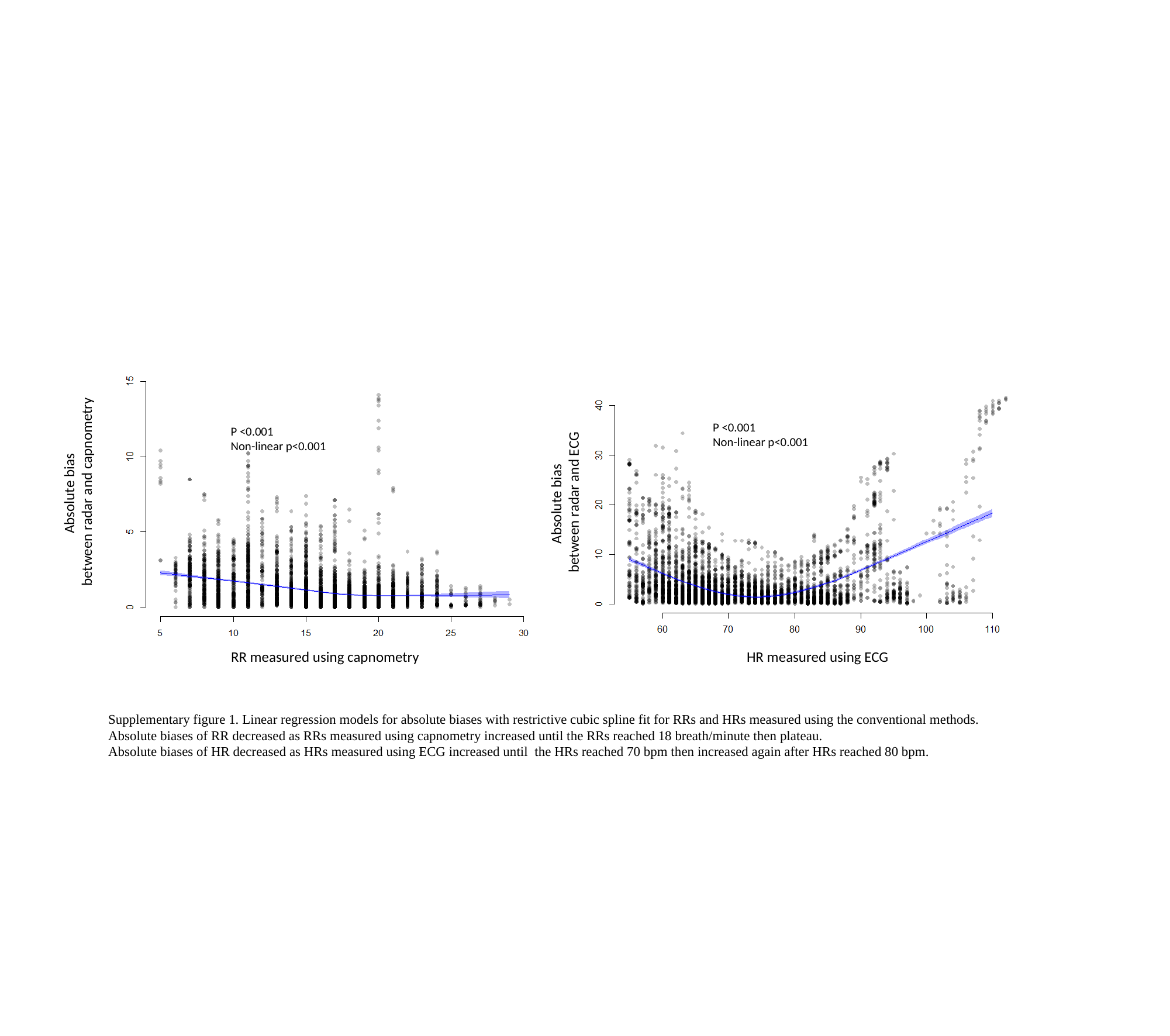

P <0.001
Non-linear p<0.001
P <0.001
Non-linear p<0.001
Absolute bias
between radar and capnometry
Absolute bias
between radar and ECG
RR measured using capnometry
HR measured using ECG
Supplementary figure 1. Linear regression models for absolute biases with restrictive cubic spline fit for RRs and HRs measured using the conventional methods.
Absolute biases of RR decreased as RRs measured using capnometry increased until the RRs reached 18 breath/minute then plateau.
Absolute biases of HR decreased as HRs measured using ECG increased until the HRs reached 70 bpm then increased again after HRs reached 80 bpm.

## Slide 2
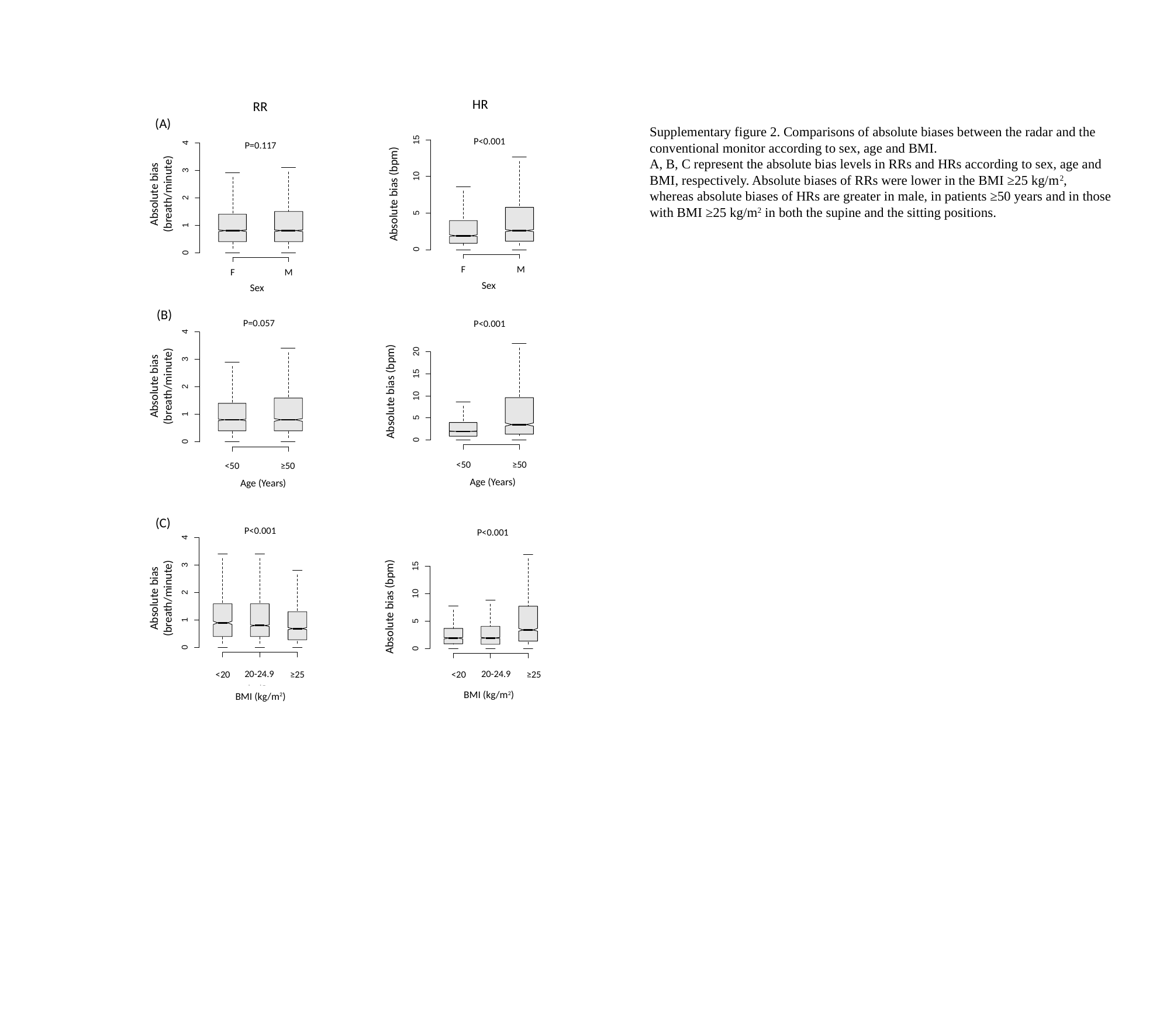

HR
RR
(A)
Supplementary figure 2. Comparisons of absolute biases between the radar and the conventional monitor according to sex, age and BMI.
A, B, C represent the absolute bias levels in RRs and HRs according to sex, age and BMI, respectively. Absolute biases of RRs were lower in the BMI ≥25 kg/m2, whereas absolute biases of HRs are greater in male, in patients ≥50 years and in those with BMI ≥25 kg/m2 in both the supine and the sitting positions.
P<0.001
P=0.117
Absolute bias
(breath/minute)
Absolute bias (bpm)
F
M
F
M
Sex
Sex
(B)
P=0.057
P<0.001
Absolute bias
(breath/minute)
Absolute bias (bpm)
<50
≥50
<50
≥50
Age (Years)
Age (Years)
(C)
P<0.001
P<0.001
Absolute bias
(breath/minute)
Absolute bias (bpm)
20-24.9
20-24.9
<20
≥25
<20
≥25
BMI (kg/m2)
BMI (kg/m2)
